# Tracking the flight and landing behaviour of western flower thrips in response to single and two-colour cues

**DOI:** 10.1038/s41598-023-37400-w

**Published:** 2023-08-30

**Authors:** Karla Lopez-Reyes, Martin J. Lankheet, Robert W. H. M. van Tol, Ruth C. Butler, David A. J. Teulon, Karen F. Armstrong

**Affiliations:** 1 Department of Pest-Management and Conservation, Lincoln University, Lincoln, 7647 New Zealand; 2 Experimental Zoology, Animal Sciences, Wageningen University and Research, PO Box 338, Wageningen, 6700AH The Netherlands; 3 Plant and Health Systems, Wageningen University and Research, PO Box 69, Wageningen, 6700AB The Netherlands; 4 Bug Research Consultancy, Herendaal 1, Maastricht, 6228GV The Netherlands; 5 StatsWork 2022 Limited, 48 Verdeco Boulevard, Lincoln, 7608 New Zealand; 6 The New Zealand Institute for Plant and Food Research Limited, Private Bag 4704, Christchurch, New Zealand; 7 Better Border Biosecurity, Lincoln, New Zealand

**Keywords:** Behavioural ecology, Entomology

## Abstract

Real-time 3D tracking and high-speed videography was used to examine the behaviour of a worldwide greenhouse pest, the western flower thrips (WFT), in response to different colours in the context of improving trap design. Measurements were taken of the number of landings on, and flight activity near, a lamp containing two LEDs of either the same colour or a combination of two colours presented side by side. Main findings show that landing patterns of WFT are different between colours, with landings on UV(+ red) as highly attractive stimulus being mostly distributed at the bottom half of the lamp, while for yellow also as very attractive and green as a ‘neutral’ stimulus, landings were clearly on the upper rim of the lamp. Additionally, a positive interaction with the UV-A(+ red) and yellow combination elicited the highest number of landings and flight time in front of the LED lamp. Conversely, a negative interaction was observed with decreased landings and flight time found for yellow when blue was present as the adjacent colour. Overall, differences between treatments were less obvious for flight times compared to number of landings, with tracking data suggesting that WFT might use different colours to orientate at different distances as they approach a visual stimulus.

## Introduction

Western flower thrips (WFT) (*Frankliniella occidentalis* Pergande) is a cosmopolitan, economically important polyphagous pest insect, impacting mainly greenhouse and some field crops by transmitting tospoviruses. Monitoring for WFT by its attraction to coloured sticky traps, in conjunction with chemical lures, capitalises on a natural behavioural response to colour while searching for potential host plants^1^. The WFT is known to show preferences to specific colours indicating colour discrimination capabilities, having a high response to blue and yellow and sometimes to UV-A light^2^. From the few electroretinogram (ERG) studies that have been conducted on WFT eyes, sensitivity peaks are found at ~ 360–365 (UV-A light)^3^, and at ~ 500–540 nm (green light)^3, 4^, which is consistent with the presence of UV-A and green photoreceptors. Behavioural studies also suggest that a blue photoreceptor is present^5^, which would increase the number of colours WFT is able to perceive and discriminate. However, there is ongoing debate regarding the most attractive colour to optimize WFT catches, especially between blue and yellow^2, 6^. To date, much of the research on WFT trapping is based on measuring the number of landings on a target^2,7^. This end-point data quantifies the overall efficacy of colour cues but provides no information about how their behaviour is influenced when approaching visual cues. Therefore, the contribution of processes leading to the outcome of targeted flight and landing is unaccounted for. At a fundamental level, the different stages of flight behaviour in close proximity to a visual cue and subsequently landing on it might use sensory inputs differently, as well as separate sensory-motor control pathways. Appreciating what influences there may be on these different aspects could better inform on the role of colour in complex environments, potentially contributing to optimization for improved monitoring of very low population densities or to progress control through mass trapping. A means to observe these aspects separately by tracking WFT flight and landing behaviour as they advance towards and then subsequently land on a visual cue would therefore be useful.

Tracking of insects under different experimental conditions has enabled the collection of important behavioural data^8, 9^, helping to detect and categorise host finding behaviour over time in a reliable, accurate and consistent manner^10^. For thrips, Thoen *et al*.^11^ used two-dimensional tracking of thrips walking on surfaces, but the 3D tracking required for flight behaviour has not been reported. Recent advances in both hardware and software have enabled successful studies of flight behaviour in a large variety of insects, ranging from bumble bees^12–14^ and honeybees^15^, to smaller insects like mosquitos^16–18^ and *Drosophila* flies^19–21^. Improvements include the real time, concurrent tracking of multiple insects achieved with high-speed video recordings^22^ and data storage capacity that is no longer a limiting factor with only the flight parameters of location and speed saved. This simultaneous, multi-object detection system enables efficient use of tracking in large-scale studies suited to quantifying flight behaviour in response to a wide range of stimulus conditions. 

Here, we study landings and approach flights separately by real-time tracking of thrips in 3D in the vicinity of a coloured LED lamp with controlled wavelength(s) and intensity. A key objective was to test real-time, multi-object, 3D tracking and high-speed videography with insects as small as WFT (~ 1–2 mm long). For this, a dedicated system was developed to meet the challenges of tracking and quantifying free-flying WFT, including optimizing the detection sensitivity for tiny targets, fast video processing to enable real-time performance at high frame rates, and reliable detection against both a back-illuminated LED lamp surface (the target) and a dark background (surroundings). The tracking system was used in a wind tunnel similar to end-point studies performed previously to study the efficacy of different colours to attract thrips^7, 23^. As a result of those studies, the emphasis here was on behaviour towards the highly attractive yellow and UV. These were used both as single colours and also in combination with each other and other colours, given natural environments are typically visually complex and may influence thrips behaviour^5^. Unlike the previous end-point analysis tracking allows the possible interactions of colours and their differential effects on approaching flights and landings to be observed. In a first experiment, the response of WFT to yellow (587 nm peak wavelength), blue (470 nm) and green (533 nm) was evaluated either as single-colour or colour combinations with the respective LEDs side-by-side in two halves of a LED lamp. In a second experiment, a UV-A light (369 nm) was included with yellow and green to test the hypothesis, based on earlier observations^7^, that UV-light has a role in the landing of WFT on visual cues.

## Materials and methods

### Insect rearing preparation

WFT were obtained from a laboratory colony kept at Wageningen University and Research (The Netherlands), reared on chrysanthemum plants (*Chrysanthemum morifolium* Ramat.) with yellow flowers, and was the same as used in previous studies^7, 23^. Rearing was at 25 °C, relative humidity of 60–70%, lighting with Philips GreenPower LED Interlighting production module (Philips Electronics, Eindhoven, The Netherlands), and a 16:8 light: dark period. For each trial 20 adult female WFT of unknown age were collected into a plastic petri dish painted with non-reflective black paint and held overnight without food for ~ 18 h. A few drops of water trapped within the two layers of stretched parafilm used to cover the petri dish gave access to water for drinking^24^. Immediately before each trial, the petri dish was put on ice for ~ 10 min to slow the insects down and keep them contained whilst moving to the release platform, opening the parafilm for release, closing the wind tunnel, and start the video recording.

### Wind tunnel and LED lamp

Experiments were run inside the wind-tunnel^7, 23^, measuring 3.0 m (l) × 1.30 m (w) × 0.7 m (h), with the test arena located in the middle. The glass ceiling was covered with a transparent polyethylene diffusing sheet (Suncover Nectarine C-980, Ginegar Plastic Products Ltd., Israel) to allow ambient lighting from above. Side walls were transparent, and the floor plus upwind and downwind screens were black. Wind speed was set at 0.3 m/s, temperature at 26 °C and RH at 65–70%. Ambient lighting within the wind tunnel was provided by LED strips on the ceiling over the test arena. This had a light spectrum component in the visible range of 400–750 nm (LED-strip–Full-colour RGB + Warm White – 24 V High Power Protected 5050, LuxaLight, NL) and a component in the ultraviolet light (UV-A) range of 360–390 nm (strip of UV LED Engin, LZ4-04UV00, Osram Sylvania Inc., US). The amount of UV-A was set as 2% of the visible light to approximate that found in natural sunlight.

**Figure 1:**
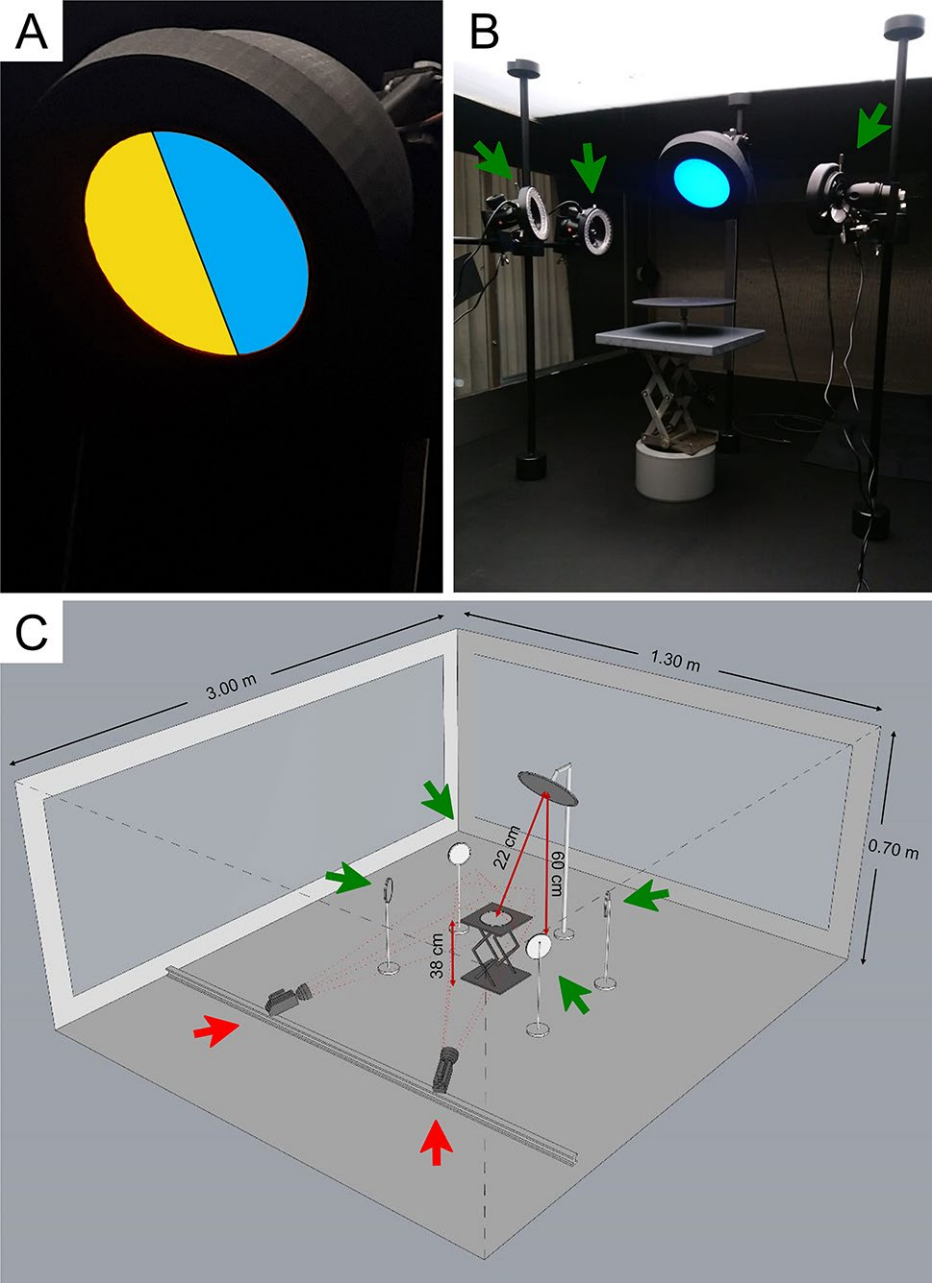
Apparatus used for Experiments 1 and 2 illustrating (**A**) close up of the LED lamp showing a two-colour treatment of yellow and blue, (**B**) view of the apparatus arrangement inside the wind tunnel with the LED lamp showing a single-colour treatment and release container in the centre, and (**C**) a sketch of the wind tunnel dimensions and apparatus placement and distances with the LED lamp and release container at the centre, the two stereoscopic high-speed cameras (red arrows) and four infrared (IR) LEDs (green arrows) used to improve detection of thrips moving in the presence of a UV-A treatment LED. Panel C was generated using Rhino 7 (https://www.rhino3d.com/).

The visual stimulus and LED apparatus used (Fig. 1A,B) is similar to that in a previous study^7^, consisting of a black, 3D-printed dome-shaped lamp case (18.5 cm diameter) with LED bulbs inside to illuminate a light-diffusing glass plate (Edmund Optics Ltd., York, UK) glued to the circular opening with an effective area of 103.86 cm^2^. A central divider split the lamp in two halves, each of which accommodated up to two LED bulbs to enable mixtures of LED lights on both sides of the lamp. Aluminium foil covering the inside of the lamp was used to homogenize the light intensities. The emission spectra and intensities for the LED ceiling strips and LED bulbs in the lamp were measured and adjusted inside the wind tunnel using a broadband spectroradiometer Specbos 1211UV (JETI Technische Instrumente GmbH, Germany).

The centre from the LED lamp was positioned at 60 cm from the wind tunnel floor and the release container was positioned at 38 cm at an angle of 45° facing down and in the downwind direction. The 45-degree tilt angle was adopted to avoid reflections of the ceiling from the lamp surface, that might interfere with the light coming from the LED lamp^23^. The release platform was located 22 cm downwind relative to the centre of the lamp (Fig. 1B,C). For each experiment, the petri dish containing the insects was placed in the middle of the release platform.

### Tracking

The tracking system consisted of two high-speed USB cameras (Basler ACE 2048-90 NIR, 2048 × 2048 pixels). The cameras were placed downwind of, and centred on, the middle of the lamp to cover a field of view of ~ 30 × 30 cm at the lamp surface (Fig. 1C). Two infrared (IR) LED rings emitting 850 nm light (60 mm Adjustable Ring Light Microscope illuminator for Industrial Camera Microscope Infrared 850/940 nm, ESHINEY Professional Optical Trading Company Store) on each side of the release platform (Fig. 1C) were used to illuminate the test arena. IR lights allowed optimal detection of thrips without disturbing them, as they are not sensitive to 850 nm light^3, 4^. In this arrangement WFT were visible as a dark shadow when flying in view of the lamp and as bright objects against the dark background of the wind tunnel.

Thrips detection and 2D tracking in both camera images was done in real-time, with no storage of video recordings necessary. Thrips flight paths in each camera view were saved for off-line reconstruction of 3D flight paths. Video was run at 85 frames per sec, tracking thrips for 15 min.

**Figure 2:**
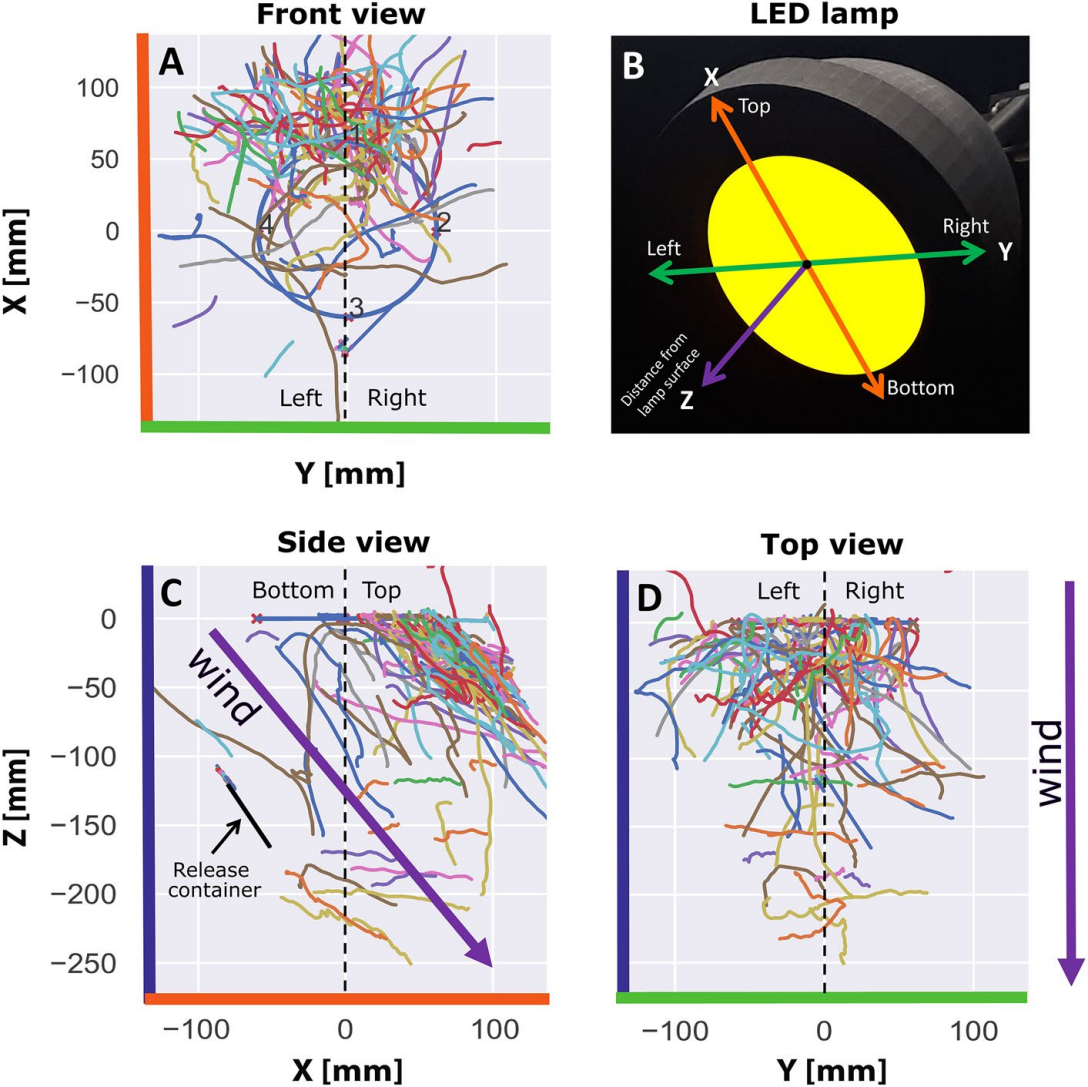
Example of recorded tracks in three different views for one trial of a single-colour yellow stimulus. X, Y and Z coordinates are defined relative to the centre of the lamp (panel **B**). (**A**) Y-axis representing left–right, (**C**) X-axis defines top–bottom and (**D**) Z-axis represents the distance relative to the lamp surface, with the blue circle indicating the circumference of the illuminated part of the LED lamp in panels (**A**, **C** and **D**). Twenty thrips were released and tracked for 15 min and each track is represented with a different colour. Purple arrows indicate the wind direction (0.3 m/s) relative to the lamp orientation 45 degrees downward in the down-wind direction. The release container is shown as a black line from the ‘side view’ of the lamp (panel **C**) and was located 22 cm from the centre of the lamp. Panels (**A**, **C** and **D**) were generated using Python 3.10 (https://www.python.org/).

For 3D reconstruction of flight paths, the cameras were calibrated as a stereo-pair, taking lens distortions into account. For calibration of the two cameras, a black and white checkerboard (6 × 8 squares, 15 × 15 mm each) was placed at random orientations and positions in view of both cameras. Checkerboard corners were automatically detected and resulting grids used to quantify lens distortions and relative camera views. Four additional calibration points placed on the rim of the LED lamp were used to link the camera coordinate system to the real-world coordinates of the setup. Data is presented in the coordinate system relative to the LED lamp (Fig. 2) where the origin corresponds to the middle-centre of the lamp, with top–bottom defined as the ‘x’ direction, ‘y’ as the left–right direction and ‘z’ as the distance orthogonal to the surface of the lamp. Because the lamp was facing 45 degrees downward, the ‘xyz’ coordinates are rotated relative to an earth-bound reference frame of the wind-tunnel.

The software used to calibrate the cameras and track the thrips was written in Python with OpenCV for fast image analysis. Detection procedures were specially optimized for thrips, including dynamic background subtraction, median filtering and a difference-of-gaussians filter optimized for the size of the insects. For object matching from frame to frame and 3D reconstruction of flight paths we used the Hungarian linker and Kalman filtering highly similar to previously reported tracking solutions^8, 22^. To exclude any data loss in case image processing could not keep up with image acquisition, images were temporarily stored in memory.

### Parameters measured in behavioural experiments

The cameras provided a limited, total view of the space around the lamp (~ 30 × 30 cm). So, individual thrips could reappear multiple times in the field of view of the cameras. Therefore, as measuring the number of tracks was not informative, we focussed on response parameters that were independent of thrips identity, including number of landings, locations on the lamp surface and total flight times in view. Also, as the cameras were located below the take-off platform, part of the platform was invisible, making it impossible to reliably track thrips take-offs from the platform. However, direct flights from the take-off platform to the lamp were rarely observed by eye, so exclusion of platform take-offs from the analysis was not considered a concern.

The total number of landings on the LED lamp per run were calculated, as well as the position of the landing sites. Landings were detected as a decrease in flight speed below the specified threshold of 3.2 cm/s, in combination with close proximity to the lamp surface and a maximal radius of 8.6 cm from the centre of the lamp. The speed-threshold was chosen based on speed-curves as a function of time and as a function of distance to the lamp. Flight times were quantified as the total sum of thrips in flight during a trial in a particular volume. To exclude thrips moving on the lamp surface we used a flight speed threshold of 6.4 cm/s, which is a factor of two times higher than that used for detecting landings.

### Behavioural experiments

Two experiments were conducted. For both, the coloured stimulus consisted of a colour on the left and on the right side of the lamp (Fig. 1A,B), which could be the same (a single-colour stimulus) or a pair of different colours (a two-colour stimulus). All LEDs were adjusted to emit light of equivalent intensity by adjusting the spectral radiance (which does not change with distance)^25^, regardless of light colour, unless stated otherwise. Except for the different combinations of LED lights (i.e., colour treatments), all procedures and light settings were equal for the two experiments. A trial consisted of total, 15 min tracking data for the 20 adult WFT released. In between trials, thrips were carefully removed from the wind tunnel. Spectra of all LEDs used for the two experiments can be found in Supplementary Figure S1.

**Experiment 1 **(December 2018) aimed to investigate the behavioural response of WFT to yellow, blue and green LEDs, comparing one- and two-colour combinations for a total of six treatments (Table 1), each replicated 16 times.

**Table 1:** Colours in Experiments 1 and 2 presented as single-^*^ or two-colour^Ϯ^ combinations, with response parameters evaluated statistically only for the target colour. Peak wavelengths of the colours used: blue (470 nm), green (533 nm), yellow (587 nm), UV (369 nm) and red (720 nm).

	Target colour	Adjacent colour
Experiment 1	Blue	Blue*
		Green^Ϯ^
		Yellow^Ϯ^
	Green	Blue^Ϯ^
		Green*
		Yellow^Ϯ^
	Yellow	Blue^Ϯ^
		Green^Ϯ^
		Yellow*
Experiment 2	UV(+ red)	UV(+ red)*
		Green^Ϯ^
		Yellow^Ϯ^
	Green	UV(+ red)^Ϯ^
	Yellow	UV(+ red)^Ϯ^
		Yellow*
	Yellow(+ red)	Yellow(+ red)*
	Red	Red*

**Experiment 2 **(February 2020) focussed on behaviour related to UV-A light, included combinations of UV-A, green and yellow, both as single colours and in combinations of UV-A light with either yellow or green. Yellow was chosen as a known, positive (attractive) stimulus and green as a neutral stimulus based on results from Experiment 1. Because the cameras used for tracking the insects were not sensitive to UV-A light, to assure identical tracking performance for all LEDs, a red LED (720 nm peak wavelength) was used inside the lamp in conjunction with the UV-A light. This provided equal excitation for the camera to maintain equal visibility of thrips in front of the UV-A light. Although 720 nm is at the limit of WFT light sensitivity^3, 4^, and therefore unanticipated to have a behavioural effect, two treatments of single-colour red light and yellow combined with red light were included as negative and positive control treatments, respectively (Table 1). The overall intensity of the LEDs, not taking red light contributions into account, was kept constant and similar amongst treatments. The intensity of the red LED was set to half the value used for all other LEDs when in combination with yellow, to prevent saturation of camera images. In total, six treatments (Table 1) were evaluated with 12 replications of each.

For both experiments, the order of stimuli was determined with an unresolvable row-column design using CycDesign 5.1 (VSN International Ltd 2013). This ensured a randomized, but balanced sequence of treatments across repetitions, minimizing potential sequence and time-of-day effects. For each colour combination, the orientation (left versus right side colour) of the first replicate each day was randomized and the second replicate was conducted in the opposite configuration.

Response parameters were quantified for each half of the lamp separately. A single treatment thus provided two measurements, one for each side of the lamp that could be considered as separate ‘target’ colours (Table 1). This allows a measurement to be analysed by its ‘target’ colour on one half of the lamp (irrespective of side), ignoring the measurement for the ‘adjacent colour’ (Table 1). Because results for single-colour stimuli and two-colour stimuli are quantified in exactly the same way (i.e., per one half of the lamp), the results for each colour stimulus can be directly comparable.

### Statistical analysis

The target and adjacent colours within each target colour were included as fixed effects in the statistical models. Initial analysis showed that side of the lamp, i.e., left or right, in which each colour was presented, and the interactions associated with it were negligible. Thus, ‘side’ as a variable was left out of the models. The variable ‘run’, i.e., each replicate of the six treatments, was included as a random effect based on various mixed models fitted for all analyses.

The number of landings (count data) was analysed using a Poisson-gamma hierarchical generalized linear model^26^). Fixed effects were fitted using a Poisson distribution and a log link, the random effect was fitted using a gamma distribution and a log link with dispersion was estimated. Importance of random effects was evaluated using a χ^2^ test assessing the change in deviance on dropping the term, as implemented in Genstat’s HGRTEST procedure. Fixed effects including particular contrasts between treatments were assessed similarly with HGFTEST. Test statistics and p-values for comparisons conducted are given in Supplementary material, Tables S3–S7. These tables include test results that were not all directly referred to in the results section, but which helped interpret the results. Subscripts on p-values below refer to test statistic tables found in Supplementary material). For flight time, times of zero were excluded since there is a qualitative difference between flying and not flying. The percentage not flying was examined (details not included) but was similar for all treatments (*p >* 0.4 for both years _p1_). Non-zero times were analysed using restricted maximum likelihood (REML)^27^. The times were log-transformed before this analysis to stabilise the variance. The fixed effects and treatment contrasts were assessed with F-tests, using the Kenward and Roger^28^ estimate for the denominator degrees of freedom. For number of landings and flight time, means were obtained using the link or transformed scale, along with 95% confidence limits. These were then converted to the original scale. All analyses were carried out using Genstat^29^.

## Results 

### General observations

On average, for the two experiments, 6.8 thrips out of the 20 released remained alive inside the release container at the end of each 15 min run, whereas 1.3 (Experiment 1) and 0.4 (Experiment 2) thrips were found dead on average inside the release container. This was used as an indication that there was negligible effect of the experimental set up to influence thrips not responding to the stimulus.

An example of the recorded tracks, illustrated here for a single trial of single-colour yellow treatment, the XYZ coordinate system relative to the lamp was defined (Fig. 2B). The side view shows that most thrips approach the lamp in the upwind direction and at the upper side of the lamp. Direct flights from the landing platform, located 22 cm from the centre of the lamp, were rarely observed. Generally, only part of the approaches led to landings; in many cases fly-bys can be seen without landings. Because no sticky glue was used on the lamp, tracks may comprise both arrivals and departures from the surface of the lamp.

**Figure 3:**
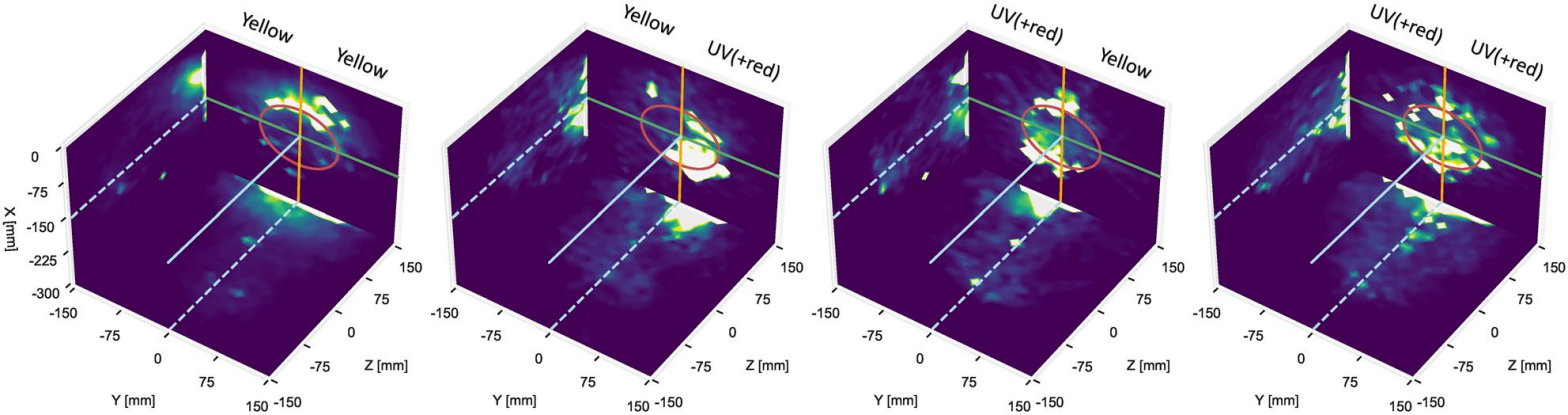
Examples of density maps of WFT activity in 3D for single-colour stimuli yellow and UV(+ red), and their colour combinations for all repetitions of each treatment. Densities reflect the total sum of thrips present in a volume element, averaged across all repetitions of a treatment. They are colour coded on an arbitrary scale and adjusted to clearly show the differences in activity in front of the lamp. The three-dimensional distribution of densities is shown as three projections onto the sides of the measured volume in front of the LED lamp. The red circle shows the illuminated part of the lamp with LEDs. Green, orange and blue lines correspond to the axes of the coordinate system as defined in Fig. 2. Densities are shown in arbitrary units, scaled similarly across the panels. Name of colours on each side of the lamp are shown on top of each treatment. This figure was generated using Python 3.10 (https://www.python.org/).

An example of the density maps is shown in Fig. 3, representing thrips averaged over all repetitions for four treatments of single- and two-colour combinations of yellow and UV(+ red), which are colours that generated the highest number of landings and flight activity. There were clear differences in activity on the lamp. For single-colour yellow, the activity is concentrated on the top side and in front of the lamp, but for single-colour UV(+ red) activity is concentrated on the lower side, with flight activity in front of the lamp more homogeneously distributed. The two-colour combination treatments (Fig. 3, middle panels) show largely increased flight activity in front of the lamp for the UV(+ red) side and reduced activity for the yellow side. These examples show that different colours, and different combinations of such colours affect landing behaviour, activity on the lamp and flight activity in front of the lamp differently.

More specifically below, we first consider the number and location of landings on each half of the lamp, as a comparison to previous end point data literature, and then assess data on flight activity as thrips densities (per volume element and per time) measured in the space in front the lamp to understand the spatial influence of colour during flight. The landing behaviour is described in more detail in relation to the attractiveness of different colours and colour interactions, and then show how flight activity in front of the lamp differs from the landing data.

### Landings

**Figure 4:**
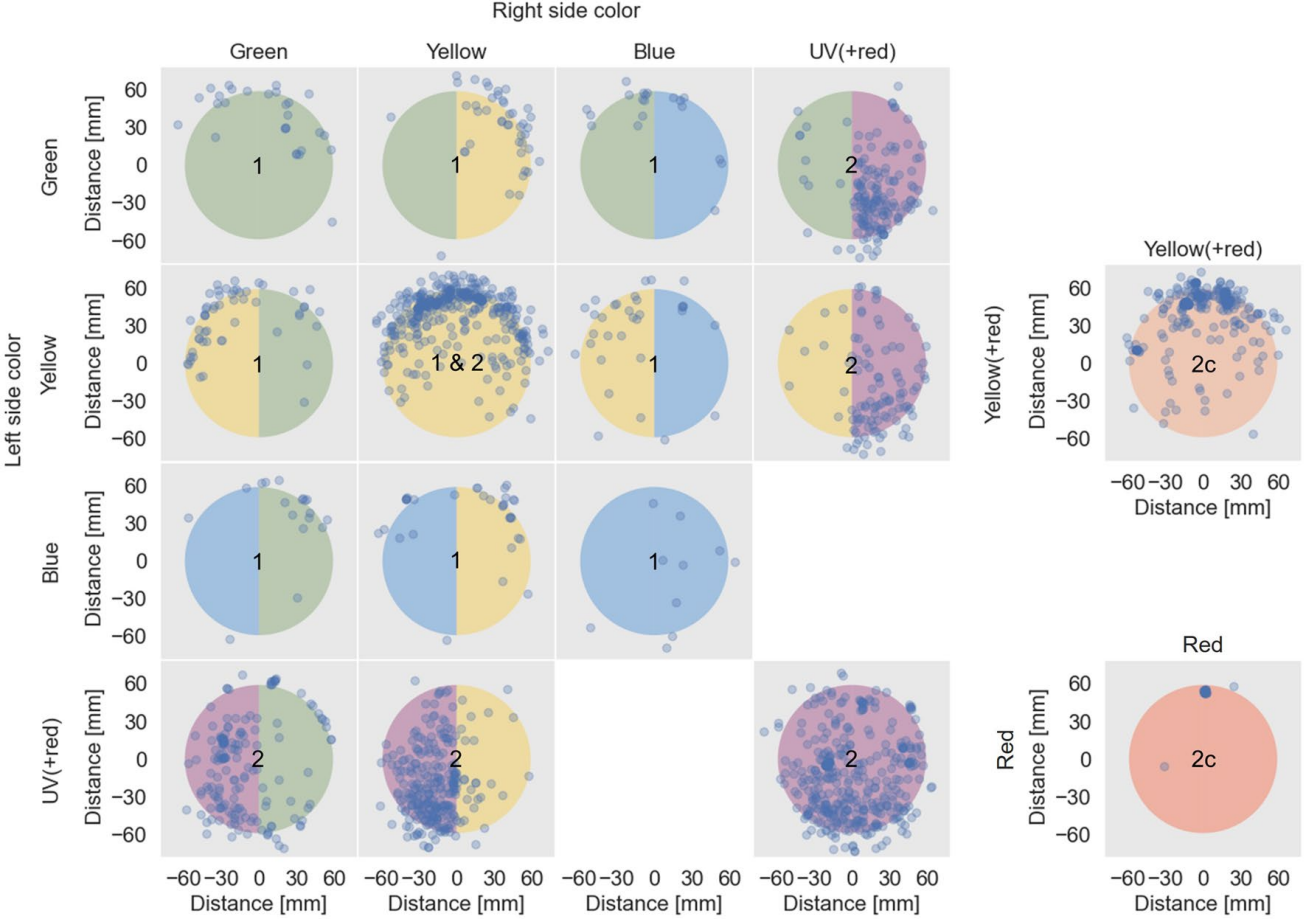
Landing sites across the 60 mm radius of the lamp for all LED colour combinations. Data points represent raw data for all treatments in both the Experiment 1 combinations for green, yellow and blue (each treatment n = 16) and Experiment 2 combinations for UV(+ red), yellow, green and red (each treatment n = 12). Yellow-yellow was included in both experiments, so effectively repeated 28 times. The experiment number for each treatment is noted in the centre of the circles. The right-hand panels denote control treatments that included red light in Experiment 2 (2c) with landings on a lamp presenting yellow light in combination with red light (yellow(+ red), n = 12) (top) and with only red light (n = 12) (bottom). This figure was generated using Python 3.10 (https://www.python.org/).

Spatial distribution of WFT landings on single- and two-colour combination treatments, for all repetitions of Experiment 1 and 2 colour treatments are shown in Fig. 4. In this qualitative assessment, apparent number of landings cannot be directly compared amongst treatments (separately analysed and described in the following section) due to the experimental design (see caption in Fig. 4 and methods section). Landing patterns were most distinct for yellow, either as a single-colour or in combination with green or blue, where WFT tended to land near the uppermost rim of the lamp. This was not so apparent when yellow was paired with UV(+ red) indicating an interaction between these colours. Similarly, green on its own or in combination with yellow or blue concentrated towards the top, but again was not so clear in combination with UV(+ red). Blue is difficult to assess with relatively few landings. On UV(+ red), landing patterns were not as distinct as with yellow but, either on its own or in combination with green or yellow, are concentrated towards the lower half of the lamp.

Two control treatments (Figs. 4, 2c), that were included to test for any effect of the red light required to detect the insects in front of UV light, support the supposition that a red light of 720 nm does not influence WFT colour response. Firstly, the pattern of landings on yellow mixed with red light (Yellow(+ red) – Figs. 4, 2c top) is no different to that for the single yellow (Fig. 4, 1&2c), and secondly with red on its own (Fig. 4, 2c bottom), too few landed to show any trend.

**Figure 5:**
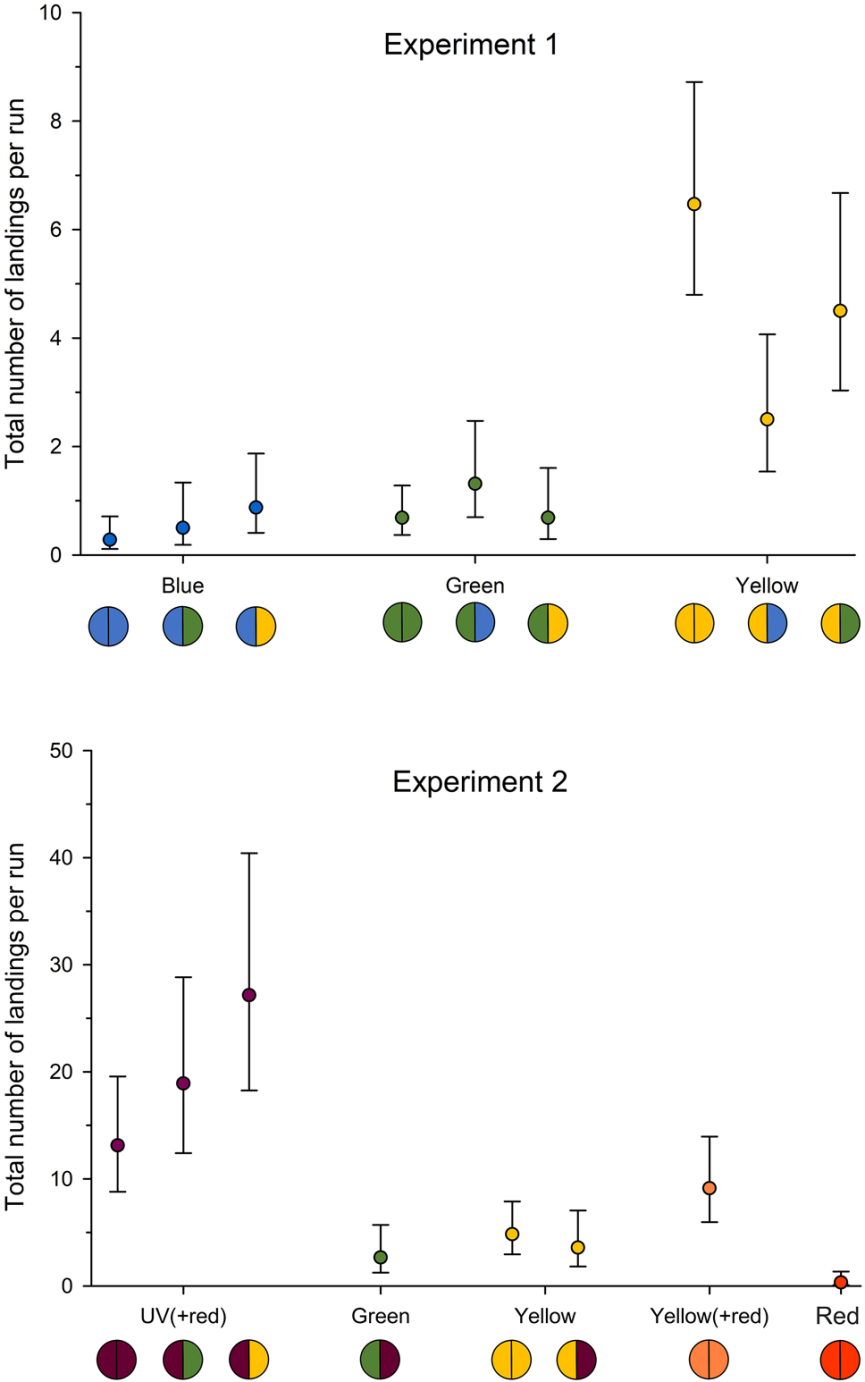
Mean total number of landings per run for the target colour shown per half of the LED lamp as listed for Experiment 1 (n = 16 repetitions) and Experiment 2 (n = 12 repetitions). Error bars are 95% confidence intervals. A table showing exact values is found in Supplementary Table S1. Below each x-axis, the left side of the coloured circle represents the target colour that was evaluated, and the right side is the adjacent colour in the lamp that was only evaluated in terms of its effect, if any, on the target colour. This figure was generated using SigmaPlot 14.5 (https://systatsoftware.com/sigmaplot/).

Regarding number of landings, the side of the lamp in which colours were presented had no statistically significant effect (*p >* 0.05 for the main effect of side for all variables presented). In Experiment 1 (Fig. 5), yellow, green and blue are shown as single-colour treatments (i.e., both sides of the lamp the same colour) as well as paired with the other two colours. Below the x-axis, the left side of the coloured circle represents the ‘target’ colour for which the measure was taken, and the right-hand side shows the paired colour, which we will refer to as the ‘adjacent’ colour. In Experiment 2 (Fig. 5), the focus was on UV(+ red) compared to yellow and green, plus the colours red and yellow(+ red) as controls for the use of red light in the UV treatment, i.e., UV(+ red). In general, the differences in both experiments were mainly related to the target colour itself, rather than to the adjacent colour. In Experiment 1, average landings varied between the colour combinations (*p <* 0.001_p2_). For single-colour treatments, yellow (6.5 landings per run) was more attractive than blue (0.7) and green (0.3) (*p <* 0.001 _p3_). For the two-colour combinations, yellow had a significantly higher number of landings per run compared to blue and green, regardless of the adjacent colour (*p =* 0.001 _p4_). However, for mean landings per run on yellow, the adjacent colour showed a substantial effect: relative to yellow being adjacent to yellow (6.5), landings were significantly lower when the adjacent colour was green (4.5), and even less when it was blue (2.5) (*p =* 0.001 _p5_, comparing between adjacent colours for yellow as a target colour). No statistically significant effect related to the adjacent colour was found for landings on either blue or green (*p =* 0.075 _p6_ and *p =* 0.266 _p7_, comparing between adjacent colours for blue and green respectively as target colours). Thus, while blue and green are not very attractive to WFT irrespective of the adjacent colour, they do have an interaction that reduces landings on yellow.

As for Experiment 1, the number of landings in Experiment 2 also varied between the colour treatments (*p <* 0.001_p8_). In Experiment 2, amongst the single-colour treatments the number of landings on yellow (as a positive control) was comparable with that in Experiment 1 (Fig. 5). Although landings on the single-colour treatment of red light in the lamp as a negative control were very low, with no evidence of being attractive, there were significantly more landings on yellow(+ red) compared to yellow without red (*p =* 0.036 _p9_). Between single-colour treatments, UV(+ red), however, showed significantly more mean landings per run (13.1) than either yellow (4.8), yellow(+ red) (9.1) or red (0.3) (*p <* 0.001 _p10_) (Fig. 5). Assessing whether red light had an influence on UV, as it did for yellow, was not possible as UV-light could not be tested in the absence of red light. For two-colour treatments, a trend of increased mean landings was found on UV(+ red) with yellow (27.2) or green (18.9) as adjacent colours, compared to the single-colour UV(+ red) (13.1) (Fig. 5). These differences were not statistically significant (*p =* 0.057 _p11_) but do indicate an interaction that could be more rigorously tested.

The two-colour treatment of UV(+ red) and yellow is interesting: UV(+ red) is overall more attractive than yellow, but yellow as the adjacent colour increased the number of landings on UV(+ red). On the other hand, the number of landings on yellow were slightly reduced when UV(+ red) was the adjacent, compared to single-colour yellow treatment (Fig. 5). These effects may indicate that UV and yellow affect different aspects of the approach and landing behaviour. As single-colour treatments they are both effective in attracting thrips, but in side-by-side combination, UV(+ red) seems more effective in inducing landings. To investigate such effects in more detail we also analysed flight activities in front of the lamps.

### Flight activity

**Figure 6:**
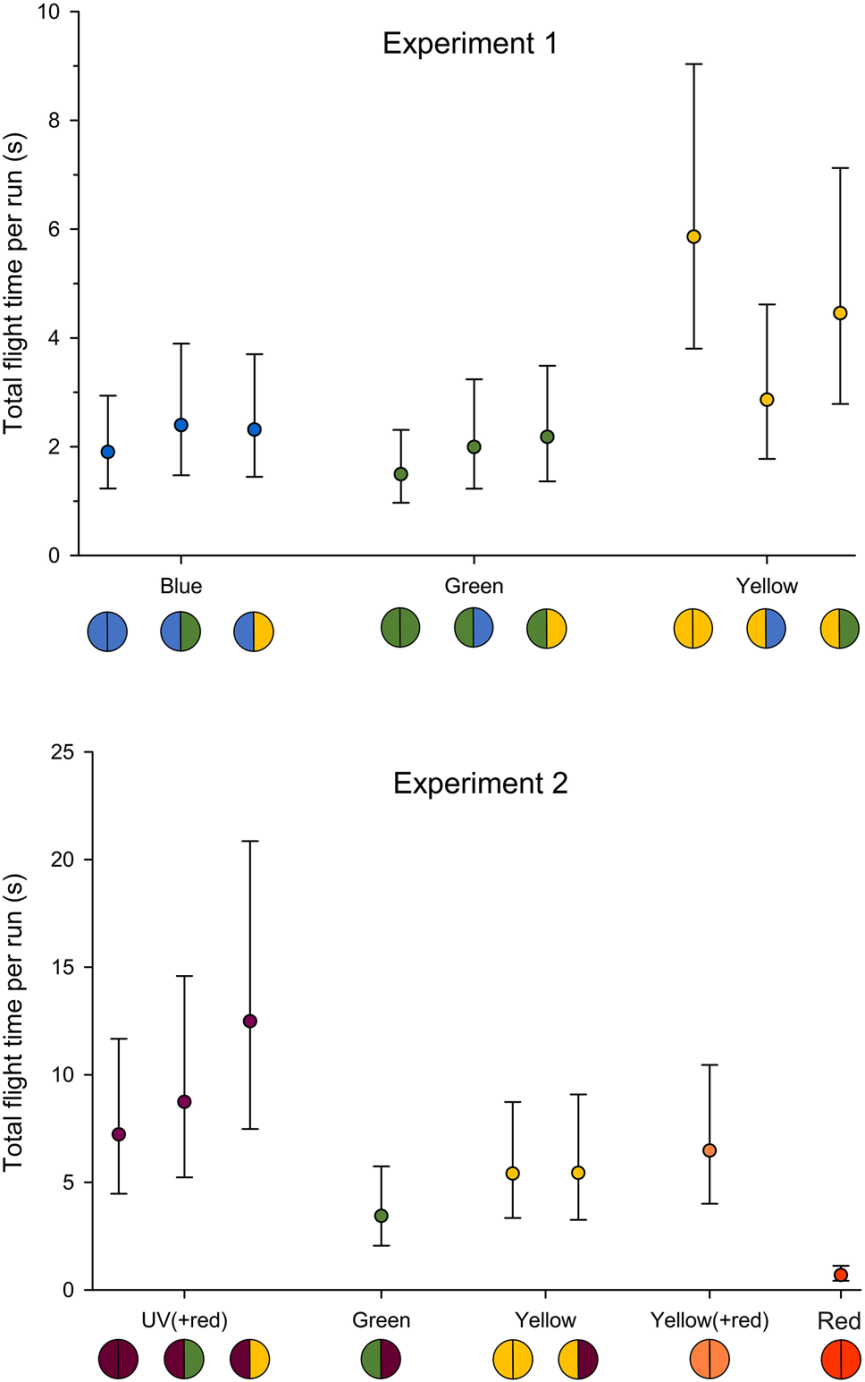
Mean total flight time (s) per run for each colour shown per half of the LED lamp as listed for all treatments in Experiment 1 (n = 16 repetitions) and Experiment 2 (n = 12 repetitions). Error bars are 95% confidence intervals. A table showing exact values is found in Supplementary Table S2. Right side of the coloured circles in the row below the x-axis is a representation of the adjacent colour tested for each of the evaluated target colours shown on the left side. This figure was generated using SigmaPlot 14.5 (https://systatsoftware.com/sigmaplot/).

As a proxy for flight activity, flight times were calculated as the sum of durations of all flights for each target colour for each half of the lamp, corresponding to flight activity on either the left- or right-hand side of the density maps (Fig. 3). Mean flight time for yellow as the positive control was again very similar between Experiment 1 (5.9 s) and Experiment 2 (5.4 s), supporting comparability between the two experiments (Fig. 6). Also, mean total flight time with the red LED alone compared to UV(+ red), green or yellow, was the lowest at 0.7 s, confirming that red light introduced into Experiment 2 had no major influence on behaviour. This was similar to the trend of red observed for landing sites (Fig. 4) and number of landings (Fig. 5). However, unlike the number of landings, there was no statistically significant differences between yellow (6.5 s) and yellow(+ red) (5.4 s) (*p =* 0.600 _p12_) (Fig. 6).

In both experiments, different colours show clear differences in flight time with only relatively minor effects of the adjacent colour. In Experiment 1, with single colour treatments (i.e., same target and adjacent colour), the mean total flight time per run was significantly higher for yellow (5.9 s) compared to blue (1.9 s) or green (1.5 s) (*p <* 0.001 _p13_) (Fig. 6). When considering the effect of the adjacent colour on the target colour, significantly higher mean flight activity was recorded for yellow when paired with yellow (5.9 s), as opposed to green (4.5 s) or blue (2.9 s) (*p =* 0.036 _p14_). No such interaction effects were found for flight activities as target colours for blue (*p =* 0.788 _p15_) or for green (*p =* 0.470 _p16_) (Fig. 6). In other words, flight times for yellow were affected by the adjacent colour, but yellow as an adjacent colour did not significantly affect flight times for blue or green.

For Experiment 2, as single-colour treatments, UV(+ red) had a significantly higher mean total flight time (7.2 s) compared to yellow (5.4 s), yellow(+ red) (5.4 s), and red (0.7 s) (*p <* 0.001 _p17_) (Fig. 6). This is enhanced for UV(+ red) when paired with green (8.7 s) or yellow (12.5 s) as adjacent colours, which is the opposite to the interaction trend observed for yellow in Experiment 1, where green and blue reduced the flight activity for yellow. However, with large variability in activity for these UV(+ red) pairings, the differences were not statistically significant (*p =* 0.300 _p18_). Interestingly, the interaction effects between UV(+ red) and yellow are clearly asymmetric: yellow as adjacent colour to UV(+ red) causes an increase in flight activity for UV(+ red), but the other way around, UV(+ red) does not affect the flight time for yellow (*p =* 0.471 _p19_).

### Flight time as a function of distance to the lamp

**Figure 7:**
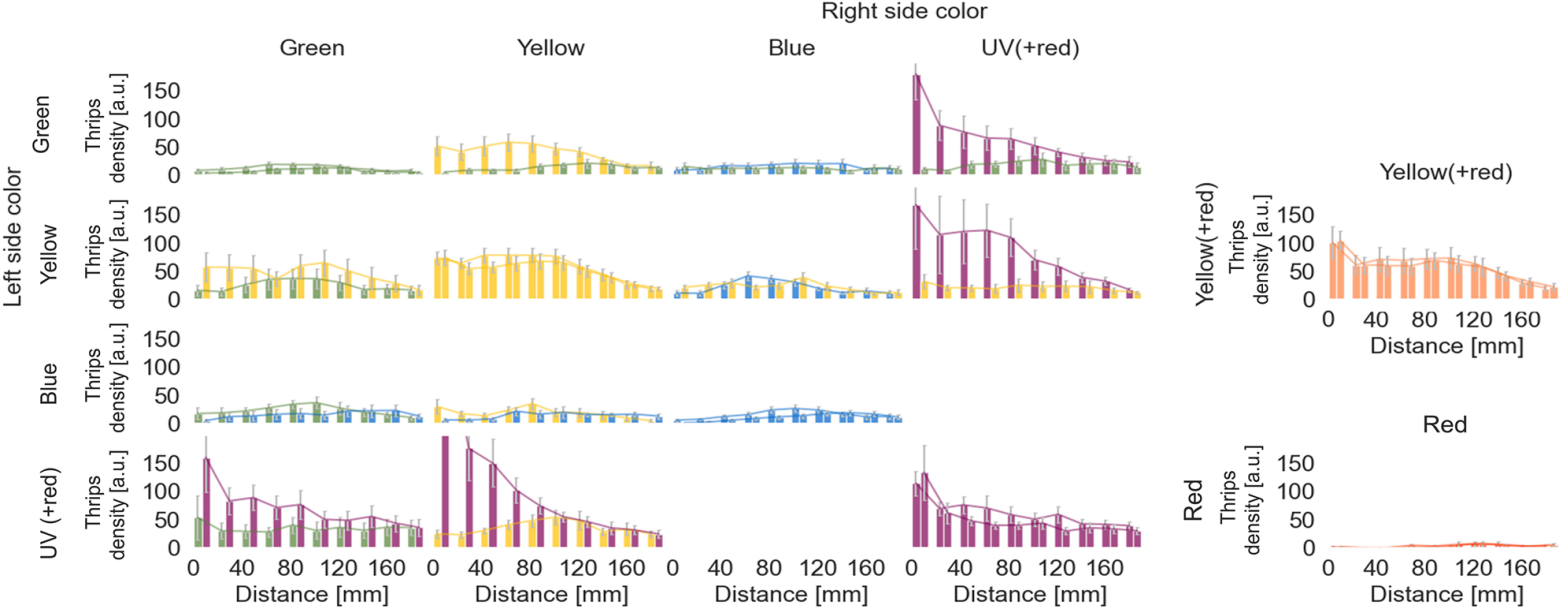
Thrips flight activity (expressed as thrips density in arbitrary units [a. u.]) as a function of distance (mm) from the LED lamp, with the colour combinations representing the two halves of the lamp. The LED lamp is exactly located at 0 mm. The distance from the lamp has been broken into 10 bins, each representing 20 mm distance. The number of runs included in each panel varies and is given in the caption of Fig. 4. Error means represent standard errors of the means. This figure was generated using Python 3.10 (https://www.python.org/).

Differences between treatments in both experiments were more pronounced for number of landings than for flight times. As an example in Experiment 2, while either green or yellow in combination with UV(+ red) as adjacent show strongly reduced numbers of landings, flight times were less affected. To explore these effects, we analysed flight times at different distances from the lamp to see at what distances these differences come into play. In comparing flight times as a function of distance to the lamp, we combined the data from treatments in Experiments 1 and 2. Flight times were calculated for 10 different distance ranges, from 0 (the lamp surface) to 200 mm in front of the lamp (Fig. 7). Flight time spent in a particular volume in front of the lamp was counted as the number of frames that a thrips was flying in such volume and is translated as thrips density (expressed as arbitrary units [a. u.]) at different distances from the LED lamp. As noted previously, yellow(+ red) is nearly identical to yellow, and red is minimally attractive. Green and blue elicit little flight activity with few flight movements across all distances and barely a maximum at intermediate distances of about 80–120 mm from the lamp. This may indicate flights that are not clearly directed towards the lamp surface, i.e., fly-bys. In contrast, for the single-colour treatments of yellow and UV(+ red), flight activity at the furthest 180–160 mm was similar to the blue or green maxima, but increased to ~ 50 a. u. by 80 mm from the lamp, staying steady at that for yellow, but with a further sharp increase for UV(+ red) to > 100 a. u. occurring at ~ 30 mm. The analysis showed the two-colour treatments to have clear distant-dependent interaction effects. The strongest interaction is observed for the pairing of yellow with UV(+ red). At distances further than ~ 100 mm from the lamp, yellow and UV(+ red) show very similar flight activities. Getting closer, however, the distributions strongly diverge with flight times in front of yellow reduced relative to the single-colour yellow pairing while at the same time flight times in front of UV(+ red) are substantially increased relative to the single-colour UV(+ red). Even though green as adjacent does not show such an influence on UV(+ red), UV(+ red) does appear to have an influence on green with consistently higher flight times even from 180 mm distance. Another obvious distance-dependent interaction is seen in the pairing of yellow and blue. When paired with blue, flight activity for yellow is considerably reduced and flight activity remains fairly constant across all distances. Collectively, the tracking information in Fig. 7, shows that some colour interactions are distance-dependent, whereas others seem unrelated to distance. This suggests that different colours may affect approaching flights and landing behaviours differently. Yellow is effective as a visual attractor, but at close range (within about 100 mm) it is nearly completely overruled by the presence of an adjacent UV(+ red).

## Discussion

There is little information about how tiny insects like WFT use colour cues to find and approach host plants. Typically, end-point data from the number of individuals caught in a trap are taken as a proxy for attractiveness, ignoring the possibility that the behaviours of attraction and landing might show different stimulus dependencies. In this study, we developed a fit-for-purpose method of real-time tracking WFT movement in 3D in the direct vicinity of an LED lamp, enabling their flight activity in that space and landing behaviour to be monitored in response to different colours and colour combinations. Despite thrips size, we were able to discern individual tracks of multiple thrips released at the same time. However, as individuals could not be identified with the level of resolution used here, the response parameters were independent of individual identity. From this, a rich dataset was generated allowing us to consider new insights into the role of colour vision in search and landing behaviour of WFT.

For both landings and flight activity UV(+ red) was the most attractive colour, followed by yellow(+ red) and yellow, with blue and green as least attractive. This is consistent with yellow and UV-A previously being found to be attractive for this WFT colony using sticky trap end-point analyses. Nonetheless, the strong response of WFT to UV-A found in this study should not be generalized, as a previous study found that different laboratory colonies of WFT from different origins can show opposite preferences to colours, such as blue and yellow^7^. Negligible attraction of WFT to the single-colour red LED (720 nm) used as a negative control was consistent with the low spectral sensitivity of WFT to 720 nm based on electroretinogram (ERG) data^3, 4^. However, we cannot rule out a small interaction effect of red light when mixed with other colours given the higher number of landings on yellow (+ red) (included as a positive control for UV(+ red)) than on yellow, although no significant difference was found in flight times. A similar comparison for UV(+ red) could not be made as the insects could not be detected with UV-light alone, but any potential interaction effect is expected to be small as UV(+ red) was substantially more attractive than either yellow or yellow(+ red).

Conclusions in the literature regarding attractiveness of WFT towards UV-A light have been scarce and mixed. Previous ERG data has shown that WFT is sensitive to wavelengths in the UV-A (~ 360–365 nm) and green (~ 500–540 nm) part of the light spectrum^3, 4^. Corresponding with the results here, some studies under controlled conditions show UV-A light elicits a high behavioural response^4, 30^, while others found only a moderate positive response^5, 31^. Only one study considered a UV-A light trap under greenhouse conditions and reported no WFT caught^30^. Nonetheless, the response of WFT to UV-A light is consistent with a sensitivity peak found in the UV-A part of the light spectrum with ERG data^3, 4^. UV-light is thought to be used by insects mainly for dispersal and migration^32–35^ and to influence and elicit flight^36^. However, here*,* UV-A light not only elicited greater flight activity, but also a high number of landings, which is not consistent with being only a stimulus for dispersal and migration. These landings could have resulted from unintentional colliding with the lamp but is unlikely given the landing site locations on UV(+ red) were not randomly distributed and mostly concentrated on the lower half of the lamp. Despite the greenhouse study mentioned above ^30^, data presented here clearly indicate the potential use of UV-light for trapping WFT.

Several colour interaction effects were identified, such as WFT attraction to UV(+ red) and yellow being influenced by the adjacent colour. The response towards UV(+ red) was higher with yellow or green adjacent compared to just UV(+ red) despite yellow and green as single colours being far less attractive than UV(+ red). The attractiveness of yellow tended to be reduced in our experiments by having green or blue as adjacent colours, especially so with blue in Experiment 1. This is in contrast to a study in *Alstroemeria* greenhouses where slightly more WFT landed on yellow sticky traps also equipped with a blue LED than yellow traps without any blue LED^6^. However, given the major methodological differences between laboratory vs greenhouse environments, LEDs vs reflective surfaces, and a laboratory colony vs wild WFT^7^, direct comparison of our results with that study are difficult. Our results do, however, resemble the effect reported by Stukenberg et al.^5^ when mixing blue and green light, suggesting a blue -green colour opponent mechanism in WFT with yellow presumably perceived by the green receptor and blue colour by a blue receptor^4, 5^. Yet, these effects most likely arise by different mechanisms, since (1) our colours were presented side-by-side (favouring spatial colour contrast effects rather than colour opponency effects) and (2) the effect found here is opposite to that of Stukenberg *et al.*^5^ in that yellow elicits a positive effect (and thus the green receptor is thought to provide a positive behavioural input) and blue has an inhibitory effect (with the blue receptor eliciting a negative behavioural response). An analogous mechanism to this has been described for migrating aphids and the pollen beetle (*Meligethes aeneus*) using behavioural data^37–40^. In contrast, similar to a phenomenon observed in whiteflies with green and UV-light mix^36^, a positive interaction was observed on UV(+ red) with yellow as adjacent colour. Thus, our results partly corroborate others in the literature, but also show a wider range of possible interactions between colours. The diversity of observed interactions shows that our findings cannot be explained by only the attractiveness of a specific colour, but that different colours most likely affect different parts of the behavioural repertoire involved in host finding. Disentangling the effects of colour contrast and colour opponency requires dedicated experiments beyond that undertaken here in which mixtures and side by side colour combinations are compared.

In addition to landings, tracking allowed us to quantify WFT flight times. Flight times between treatments were mostly explained by the target colour, with minor to moderate modulations by the adjacent colour, but less pronounced than for landings. This suggest WFT may have different strategies and use cues differently in free flight compared to when they are preparing to land. For example, UV(+ red) and yellow were more or less equally effective in attracting flights, but landing locations clearly varied when UV(+ red) was paired with yellow, as the response of WFT to UV(+ red) completely overruled that towards yellow. Differences in landing patterns by different colours might be related to spatial variability in the distributions of colour receptors in the WFT eye and the relative orientation of the insects’ head from colour cues. Our results suggest that in free flight, both UV-A and long-wavelength lights like green and yellow may be used to control flight direction. Landing control, however, seems to be driven by UV-A light, if present, and otherwise by long-wavelength light. In addition, distance-dependent interaction effects show that final landing strategies may be controlled differently compared to approach flights. For bees, approaching flights from within 5–10 cm towards single- and two-colour blue/yellow stimuli are usually from below the target when the stimulus is arranged in the vertical plane^41^. Such a strategy in approach flights can lead insects to land in specific locations of the target, such as the LED lamp used here. Our data do not allow us to assess colour preferences at longer distances, as our field-of-view was limited for technical purposes to a volume of about 30 × 30 × 20 cm. However, colour cues were shown to become less important for WFT with increasing distance in other studies^42^, which is consistent with their rather low visual acuity leading to colour differences, including colour contrast effects, becoming less distinct with increasing distance. Nevertheless, although we tried to have equivalent intensities for the different colours whenever possible (with the exception being UV(+ red) and yellow (+ red)) to remove intensity as a variable, cues at long distances may depend on the type of stimulus and its intensity relative to the background. Therefore, it remains an open question to what extent coloured LED lamps could be used for long-distance attraction, together with intensity and colour contrast.

Generally, insects can use both intensity contrast (i.e. achromatic contrast) and colour contrast for object detection^43, 44^. By adjusting LEDs to the same spectral radiance, we aimed to minimize the intensity effects as much as possible, but quantifying colour contrast and intensity contrast^45^ for WFT is difficult because, contrary to bees and many other insect species, information about individual photoreceptor spectral sensitivity remains unclear^4^. Indications that colour contrast might indeed play a role in thrips navigation is suggested from the number of WFT caught on coloured traps hung over a background of coloured-flower cultivars compared to being hung over a white cultivar^6, 46^. In our study, we cannot rule out that part of the behavioural differences resulted from achromatic visual processing. To what extent colours of equal radiance are perceived as equiluminant by the insect also depends on the relative and spectral sensitivities of the different receptor systems. Moreover, for UV-A treatments we added red light for tracking purposes, which effectively increased the overall radiance of the stimulus, although the controls here showed that it had little or no influence on our main findings. On the other hand, colour contrast or a visual edge between two colours could aid thrips to differentiate visual cues more easily and induce them to land near boundaries of visual targets, as shown with other insects^41, 44^. In our results, we found that for yellow and green, the landing sites were concentrated around the edge of the LED near the light contrast border between the LED and the black lamp frame. For UV(+ red) as a single-colour this effect was clearly absent, but next to yellow or green landings showed a slight tendency to concentrate near the border between the two colours. For other two-colour treatments, like blue and yellow or blue and green where intensities were equalized, no such boundary effects were observed. To what extent intensity contrasts plays a role on flight and landings of WFT remains unknown. It is obvious though, that in the absence of intensity contrast, colour contrast and colour vision can enable insects to detect and approach visual targets^44^.

In conclusion, using a 3D insect tracking system based on high-speed videography we were able to effectively study WFT flight and landing behaviour in the vicinity of a visual target. Beyond confirming that both yellow and UV-A light are highly effective in attracting thrips, we found that flight activity at close range and landings were quite different between these two colours, with UV-A light overruling yellow-elicited landings. Although red, green and blue were of little attraction by themselves, they had a modulating effect on attraction to UV-A and yellow. Our results suggest that distinct colour detection mechanisms maybe involved for WFT at different flight stages in response to a visual stimulus. The real-time tracking apparatus was key to this study, allowing large volumes of data-processing to be done “on the fly”, excluding the need to store and post-analyse massive amounts of video data. With some improvements and adjustments, the system could also be used to study long-distance target finding, including the influence of combining olfactory and visual cues. This would help to further elucidate the mechanism of host finding that could be applied to improved trapping systems.

### Supplementary Information

Supplementary Information.

## Data Availability

The data presented in this study are available on request from the corresponding author.
