# Supplementary material for: Tracking the flight and landing behaviour of western flower thrips in response to single and two-colour cues
Source: Sci Rep. 2023 Aug 30;13:14178. doi: 10.1038/s41598-023-37400-w (PMC10469208; doi:10.1038/s41598-023-37400-w)
Supplement: Supplementary file 1 — Supplementary Information. [file 41598_2023_37400_MOESM1_ESM.pdf]

Tracking the flight and landing behaviour of Western Flower Thrips in response to single and two-colour cues

Karla Lopez-Reyes , Martin J. Lankheet , Robert W. H. M. van Tol , Ruth C. Butler , David A. J. Teulon and Karen F. Armstrong

Supplementary information

Supplementary Table S1. Mean total number of landings per run per target colour on the LED lamp for Experiment 1 (n=16) and Experiment 2 (n=12) shown in Figure 4. Numbers in brackets are 95% confidence intervals.

	Target colour	Adjacent colour	Number of landings (95% confidence intervals)
**Experiment 1**	Yellow	yellow	6.5 (4.8,8.7)
		blue	2.5 (1.5,4.1)
		green	4.5 (3.0,6.7)
	Blue	blue	0.3 (0.1,0.7)
		yellow	0.9 (0.4,1.9)
		green	0.5 (0.2,1.3)
	Green	green	0.7 (0.4,1.3)
		yellow	0.7 (0.3,1.6)
		blue	1.3 (0.7,2.5)
**Experiment 2**	UV(+red)	uv(+red)	13.1 (8.8,19.6)
		yellow	27.2 (18.3,40.4)
		green	18.9 (12.4,28.8)
	Green	uv(+red)	2.7 (1.2,5.7)
	Yellow	yellow	4.8 (3.0,7.9)
		uv(+red)	3.6 (1.8,7.1)
	Yellow(+red)	yellow(+red)	9.1 (6.0,14.0)
	Red	red	0.3 (0.1,1.3)

Supplementary Table S2. Mean total flight time per run per target colour on the LED lamp for Experiment 1 (n=16) and Experiment 2 (n=12) shown in Figure 5. Numbers in brackets are 95% confidence intervals.

	Target colour	Adjacent colour	Flight time (s) (95% confidence intervals)
**Experiment 1**	Yellow	yellow	5.9 (3.8,9.0)
		blue	2.9 (1.8,4.6)
		green	4.5 (2.8,7.1)
	Blue	blue	1.9 (1.2,2.9)
		yellow	2.3 (1.4,3.7)
		green	2.4 (1.5,3.9)
	Green	green	1.5 (1.0,2.3)
		yellow	2.2 (1.4,3.5)
		blue	2.0 (1.2,3.2)
**Experiment 2**	UV(+red)	uv(+red)	7.2 (4.5,11.7)
		yellow	12.5 (7.5,20.9)
		green	8.7 (5.2,14.6)
	Green	uv(+red)	3.4 (2.1,5.7)
	Yellow	yellow	5.4 (3.3,8.7)
		uv(+red)	5.4 (3.3,9.1)
	Yellow(+red)	yellow(+red)	6.5 (4.0,10.5)
	Red	red	0.7 (0.4,1.1)

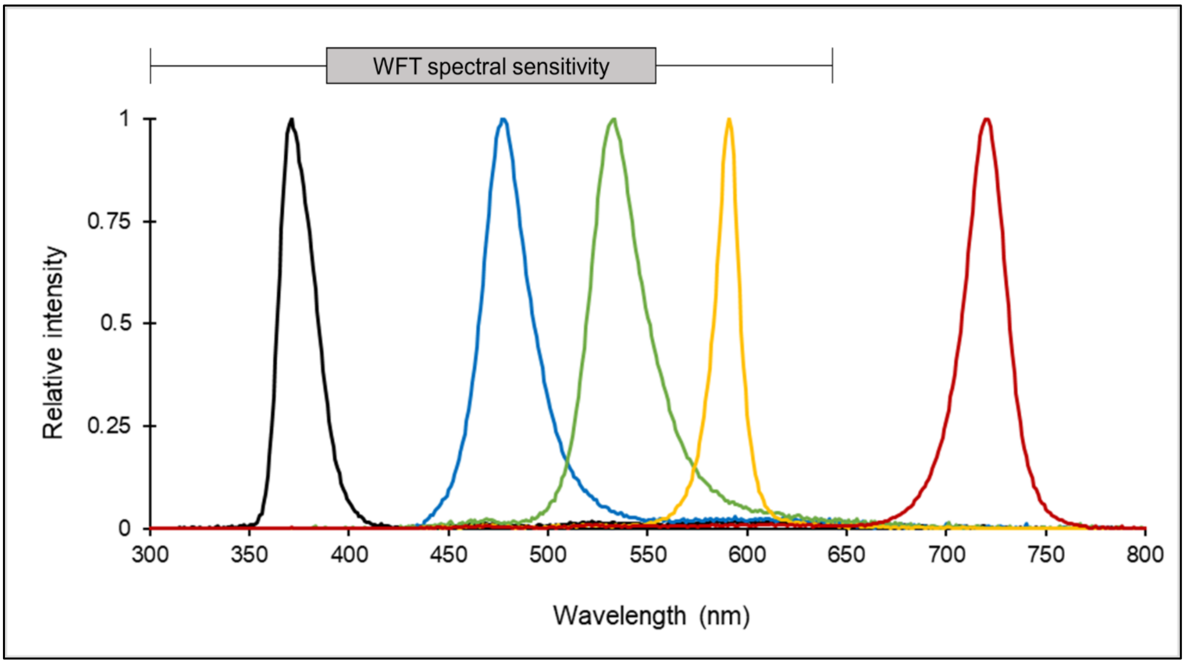

Supplementary Figure S1. Light spectra of LEDs inside the wind tunnel used for Experiments 1 and 2 with peak wavelengths of 371 (UV-A), 477 (blue), 533 (green), 591 (yellow) and 720 (red) nm.

Test statistics and probabilities for various comparisons for the two variables referred to in the main text (Total number of landings and Flight Time for time >0), plus also % of runs with Flight Time > 0. Results are for the two experiments separately. Test statistics for Total number of landings on the target colour and % of runs with Flight Times > 0 are $\chi 2$ statistics with their associated degrees of freedom. Test statistics for Flight Time for time >0 are F statistics with their associated numerator and denominator degrees of freedom. Denominator degrees of freedom are estimated with the Kenwood & Roger method and are generally not integers. Subscripts for p-values, (e.g. $_{(p1)}$, $_{(p2)}$ etc.) refer to particular p-values mentioned in the main text within the Results section. Not all p-values shown in Supplementary Tables S3-S7 are referred to in the main text. Since the percentage of runs with Flight times > 0 was largely the same for all colour pairs, further test results for this variable are not presented in the tables below.

Table S3. Test statistics for overall comparison between colours. DF = degrees of freedom.

**Variable**	**Experiment 1 (2018)**			**Experiment 2 (2020)**		
	**Test statistics**	**DF**	**p-value**	**Test statistics**	**DF**	**p-value**
Total number of landings	116.0	5	<0.001 $_{(p2)}$	64.7	5	<0.001 $_{(p8)}$
% runs with flights > 0	0.9	5	0.455 $_{(p1)}$	0.7	5, 138	0.606 $_{(p1)}$
Flight time, time > 0	4.7	5, 85.6	<0.001	14.6	5, 65.3	<0.001

Table S4. Test statistics for comparison between single-colour treatments, and between two-colour colour treatments. DF = degrees of freedom.

Variable	Experiment	Single-colour treatments	DF	p-value	Two-colour treatments	DF	p-value
Total number of landings	1 (2018)	103.1	2	<0.001 (p3)	13.0	2	0.001 (p4)
	2 (2020)	52.1	3	<0.001 (p10)	1.8	1	0.185
Flight time, time > 0	1 (2018)	11.1	2, 85.9	<0.001 (p13)	0.6	2, 85.3	0.529
	2 (2020)	21	3, 65.4	<0.001 (p17)	1.4	1, 65.0	0.233

Table S5. Test statistics for overall comparison between colours. DF = degrees of freedom.

Variable	Experiment	Target colour test	DF	p-value
Total number of landings	1 (2018)	150.1	2	<0.001
	2 (2020)	177.5	4	0.043
Flight time, time > 0	1 (2018)	15.9	2, 137	<0.001
	2 (2020)	29.3	4, 104.6	<0.001

Table S6. Test statistics for comparison between adjacent colours for each target colour. DF = degrees of freedom.

Variable	Experiment	Target yellow	DF	p-value	Target blue	DF	p-value	Target green	DF	p-value	Target UV(+red)	DF	p-value
Total number of landings	1 (2018)	14.3	2	0.001 (p5)	5.2	2	0.075 (p6)	2.6	2	0.266 (p7)	-		
	2 (2020)	0.2	1	0.661	-			-			5.7	2	0.057 (p11)
Flight time, time > 0	1 (2018)	3.4	2, 137	0.036 (p14)	0.2	2, 137	0.788 (p15)	0.8	2, 137	0.470 (p16)	-		
	2 (2020)	0.5	1, 74.7	0.471 (p19)	-			-			1.2	2, 77.4	0.300 (p18)

Table S7. Test statistic comparison between single-colour yellow and single-colour yellow(+red), Experiment 2 (2020). DF = degrees of freedom.

Variable	Test statistic	DF	p-value
Total number of landings	4.4	1	0.036 (p9)
Flight time, time > 0	0.3	1, 65	0.600 (p12)
